# Lung shrinking assessment on HRCT with elastic registration technique for monitoring idiopathic pulmonary fibrosis

**DOI:** 10.1007/s00330-022-09248-7

**Published:** 2022-11-23

**Authors:** Haishuang Sun, Xiaoyan Yang, Xuebiao Sun, Xiapei Meng, Han Kang, Rongguo Zhang, Haoyue Zhang, Min Liu, Huaping Dai, Chen Wang

**Affiliations:** 1 Department of Respiratory Medicine, The First Hospital of Jilin University, Changchun, 130021 China; 2 Department of Pulmonary and Critical Care Medicine, China-Japan Friendship Hospital, National Center for Respiratory Medicine, Institute of Respiratory Medicine, Chinese Academy of Medical Sciences, National Clinical Research Center for Respiratory Diseases, Yinghua Dong Street, Hepingli, Chao Yang District, Beijing, 100029 China; 3 Chinese Academy of Medical Sciences and Peking Union Medical College, Beijing, 100730 China; 4 Department of Radiology, China-Japan Friendship Hospital, Beijing, 100029 China; 5 Institute of Advanced Research, Infervision Medical Technology Co., Ltd., Beijing, 100025 China; 6 Department of Radiology, University of California, Los Angeles, Los Angeles, 90095 USA

**Keywords:** Image processing, Computer-assisted, Idiopathic pulmonary fibrosis, Interstitial lung disease, Computed tomography, X-ray

## Abstract

**Objectives:**

Evaluation and follow-up of idiopathic pulmonary fibrosis (IPF) mainly rely on high-resolution computed tomography (HRCT) and pulmonary function tests (PFTs). The elastic registration technique can quantitatively assess lung shrinkage. We aimed to investigate the correlation between lung shrinkage and morphological and functional deterioration in IPF.

**Methods:**

Patients with IPF who underwent at least two HRCT scans and PFTs were retrospectively included. Elastic registration was performed on the baseline and follow-up HRCTs to obtain deformation maps of the whole lung. Jacobian determinants were calculated from the deformation fields and after logarithm transformation, log_jac values were represented on color maps to describe morphological deterioration, and to assess the correlation between log_jac values and PFTs.

**Results:**

A total of 69 patients with IPF (male 66) were included. Jacobian maps demonstrated constriction of the lung parenchyma marked at the lung base in patients who were deteriorated on visual and PFT assessment. The log_jac values were significantly reduced in the deteriorated patients compared to the stable patients. Mean log_jac values showed positive correlation with baseline percentage of predicted vital capacity (VC%) (*r* = 0.394, *p* < 0.05) and percentage of predicted forced vital capacity (FVC%) (*r* = 0.395, *p* < 0.05). Additionally, the mean log_jac values were positively correlated with pulmonary vascular volume (*r* = 0.438, *p* < 0.01) and the number of pulmonary vascular branches (*r* = 0.326, *p* < 0.01).

**Conclusions:**

Elastic registration between baseline and follow-up HRCT was helpful to quantitatively assess the morphological deterioration of lung shrinkage in IPF, and the quantitative indicator log_jac values were significantly correlated with PFTs.

**Key Points:**

• *The elastic registration on HRCT was helpful to quantitatively assess the deterioration of IPF*.

• *Jacobian logarithm was significantly reduced in deteriorated patients and mean log_jac values were correlated with PFTs*.

• *The mean log_jac values were related to the changes of pulmonary vascular volume and the number of vascular branches*.

**Supplementary Information:**

The online version contains supplementary material available at 10.1007/s00330-022-09248-7.

## Introduction

Idiopathic pulmonary fibrosis (IPF) characterized by progressive and irreversible fibrosis of the lung parenchyma and respiratory failure is associated with a high mortality rate [1]. The pathology of IPF is characterized by diffuse alveolar inflammation, alveolar structural disorders, and fibrosis [2]. Pulmonary function tests (PFTs), such as forced vital capacity (FVC) and diffusing capacity for carbon monoxide (DLco), which tend to decline over time, are the most common indicators of the progression of IPF [3, 4]. Moreover, in recent years, the role of pulmonary vascular remodeling in the development and prognosis of interstitial lung disease (ILD) has received increasing attention and pulmonary vascular remodeling may be a novel imaging biomarker in addition to pulmonary fibrosis for assessing the disease severity in patients with IPF [5, 6]. Changes in pulmonary vascular structure may be related to pulmonary shrinkage due to lung fibrosis at the corresponding sites and other partial compensatory expansions. Pulmonary fibrosis lesions in patients with IPF are often accompanied by shrinkage in the same areas of the lungs and lead to changes in pulmonary vascular structure and number. Meanwhile, as fibrosis progresses, lung shrinkage progressively worsens. Alterations in pulmonary vascular structure are therefore not only associated with pulmonary shrinkage but also with the prognosis of IPF.

High-resolution computed tomography (HRCT) is the key method to evaluate ILD [7]. However, visual assessment of IPF progression on HRCT is usually subjective [8, 9]. Pulmonary shrinkage necessitates side-by-side comparison for each level of CT scans. Image elastic registration applies a variety of geometric transformations over one or more moving images in order to match and establish correspondence with a target image [10, 11]. A Jacobian map is obtained by calculating the logarithm of the Jacobian (log_jac) determinant for each voxel of the deformation field. In terms of ILD, elastic registration has proven to be useful for the evaluation and quantification of the local lung deformation in several relevant researches [12, 13]. Chassagnon et al [14] performed elastic registration on inspiratory-expiratory magnetic resonance imagings in patients with systemic sclerosis–associated interstitial lung disease (SSc-ILD). Jacobian maps of their research showed marked shrinkage at the base of the lung and with smaller log_jac in the participants without fibrosis compared to participants with fibrosis, which indicated less respiratory deformation in patients with pulmonary fibrosis. Next, they demonstrated that elastic registration on CT scans could assess disease progression during the follow-up of SSc-ILD [12]. At present, quantitative analysis of IPF with an elastic registration–based method has not been reported. The objective of this study was to evaluate lung shrinking on HRCT scans as a marker of longitudinal changes in IPF and make correlation analysis with disease progression indicators such as PFTs and pulmonary vascular–related parameters.

## Materials and methods

### Study population

**Fig. 1:**
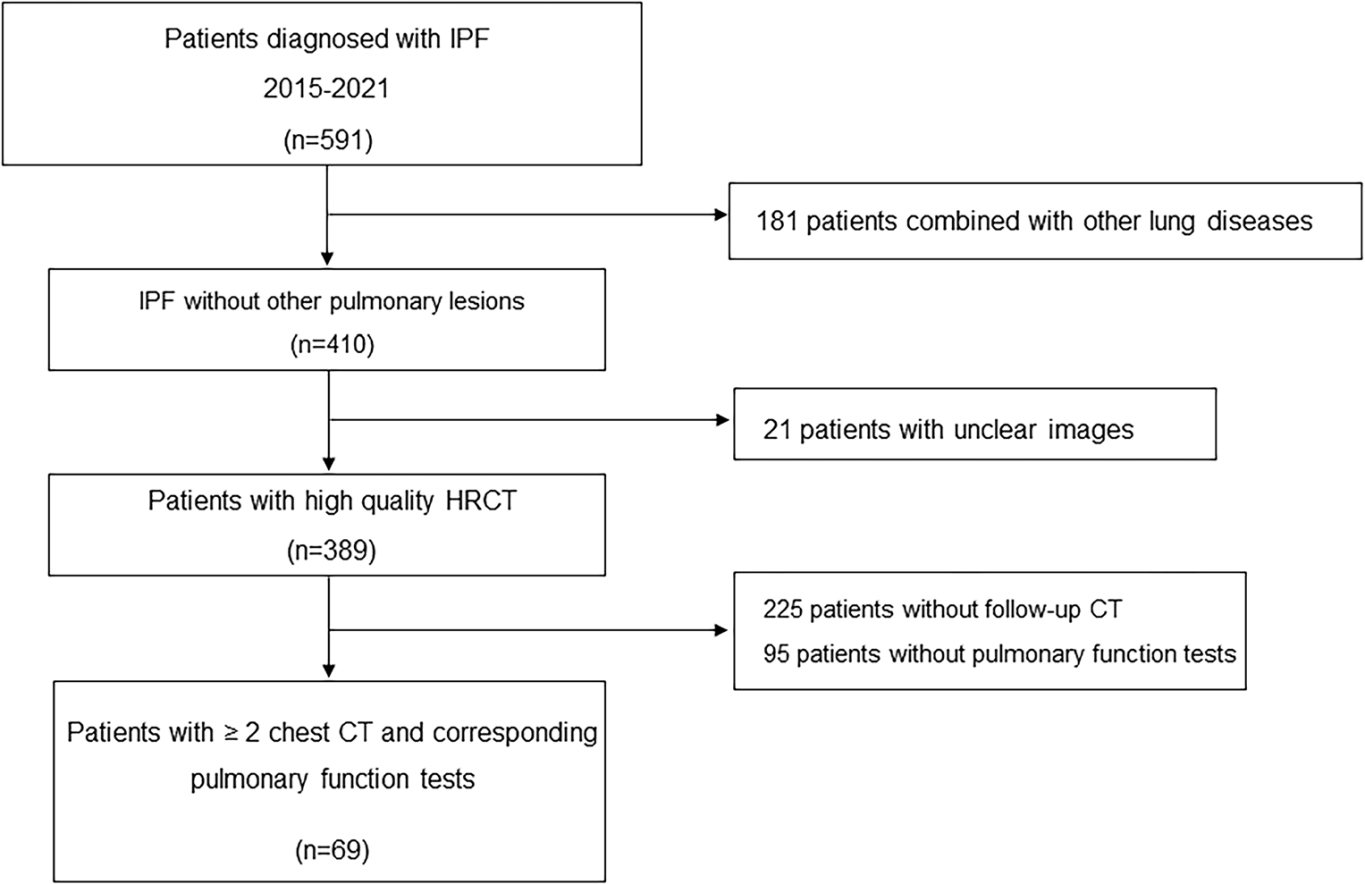
The flow diagram of eligible patients in this research

This retrospective single-center cohort research was approved by our institutional ethics committee (study number, 2017-25). In this retrospective study, consent was waived for each patient. All included patients underwent PFTs with pulmonary function test system (MasterScreen) in the same day of HRCT. Percentage of predicted vital capacity (VC%), percentage of predicted forced vital capacity (FVC%), percentage of predicted total lung capacity (TLC%), and percentage of predicted diffusing capacity for carbon monoxide (DLco%) were included in PFT measurements. Functional deterioration was defined as 10% absolute decrease in FVC% or 15% absolute decrease in DLco% assessed on the basis of the ATS/ERS/JRS/ALAT guidelines [15]. A total of 69 patients diagnosed with IPF in our hospital from January 2015 to May 2021 were included. IPF was diagnosed by the multidisciplinary team diagnosis based on the 2011 American Thoracic Society, European Respiratory Society, Japanese Respiratory Society, and Latin American Thoracic Association (ATS/ERS/JRS/ALAT) criteria [15]. The inclusion criteria were (1) patients with IPF who underwent at least two HRCT scans and (2) HRCT and the corresponding PFTs were performed on the same day. The exclusion criteria were (1) poor quality of HRCT scans with significant artifact; (2) combination with other lung disease, surgery, or pleural effusion; and (3) incomplete PFT data. If the patient underwent more than two HRCTs during the follow-up period, only the earliest and the latest HRCTs were included. Figure 1 shows a flowchart detailing how participants were selected and how the research was conducted.

### CT protocol

All HRCT scans in our study were non-contrast-enhanced CT and were performed using multidetector CT systems (LightSpeed VCT/64, GE Healthcare; Toshiba Aquilion ONE TSX-301C/320; Philips iCT/256; Siemens FLASH Dual Source CT). The whole chest was craniocaudally scanned in a supine position from the lung apex to the lowest hemidiaphragm during a single breath-hold. Acquisition parameters and reconstruction parameters were in accordance with CT standards: tube voltage of 100–120 kV, tube current of 100–300 mAs, slice thickness of 0.625–1 mm, table speed of 39.37 mm/s, gantry rotation time of 0.8 s, and reconstruction increment of 1–1.25 mm. All participants of the study were in a supine position with their hands raised above their heads to cooperate with the examination.

### Visual assessment

Visual assessment of HRCT scans was respectively performed by two radiologists (X.M. and X.S.) with 3 and 5 years of experience in chest radiology. If there was a disagreement of opinion, the final evaluation was made by the third chest radiologist with 15 years’ experience (M.L.). The clinical procedure and the PFTs were blinded for all radiologists. Baseline and follow-up HRCTs were then compared independently by two radiologists to assess whether ILD exhibited morphologic stability, deterioration, or improvement. Improvement was recorded when follow-up HRCT showed the decreased or diminished ground-glass opacities. Deterioration was reported if ground-glass opacities or traction bronchiectasis and/or honeycombing increased; otherwise, it was reported to be morphologically stable.

### Elastic registration

According to Chassagnon et al [12], all HRCTs were first preprocessed by isotropic sampling with 1 mm, and lung segmentation used by a medical imaging solution software, which provided a deep learning–based segmentation method, UNet, to perform automatic segmentation of lung fields [13]. Then, lung fields were automatically segmented by a fully automated deep learning convolutional neural network (InferRead™ CT Lung, version R3.12.3; Infervision Medical Technology Co., Ltd.). Next, elastic registration was performed via symmetric affine and deformable transformation using ElasticSyN from Advanced Normalization Tools (ANTs) [16], to align follow-up CT scans with the baseline CT scans.

Deformation field, i.e., the field composed of the displacement of each voxel when the follow-up CT volume was matched to the corresponding baseline volume, for each pair of CT scans was generated after this registration. Finally, a Jacobian map was obtained by calculating the logarithm of the Jacobian (or log_jac) determinant for each voxel of the deformation field. The Jacobian determinant is a common measurement to quantify morphological change of each voxel after registration. The voxel size after deformation is interpreted as unchanged after deformation if log_jac = 0; shrinkage, < 0; expansion, > 0.

A Jacobian map is a visual representation of the logarithmic form of the Jacobian determinant, which is also used to conduct intuitive visual comparison between patient groups split by either visual assessment or functional evaluation, which is similar to the study by Chassagnon et al [12]. Specifically, each Jacobian map was registered to a common space using the same registration method. After averaging the registered Jacobian maps for each patient group (groups of morphological improvement, stability, and deterioration according to visual assessment on CT; for groups of functional stability and deterioration according to pulmonary function assessment), a particular three-dimensional Jacobian map was available for visual comparison. The mean of the Jacobian map for each patient was also calculated for inter-group comparative analysis and PFT correlation test. All the above operations were implemented in the Python environment (version 3.5; Python Software Foundation) based on an Ubuntu operating system (version 16.04; Canonical Ltd.).

### Vessel segmentation and assessment

HRCT scans in Digital Imaging and Communications in Medicine format (DICOM) were transferred to a 3D in-home workstation (FACT AI+-digitalLung V1.0, Shenzhou Dexin Medical Imaging Technology Co., Ltd.) and pulmonary vessels were automatically segmented with an automatic integration segmentation approach [17]: (1) identifying the extrapulmonary arteries and veins using a U-Net architecture, (2) identifying the intrapulmonary vessels using a computational differential geometry solution, (3) skeletonizing the intrapulmonary vessels, which guides the tracing of neighboring vessel branches, and (4) tracing the skeletons of the intrapulmonary vessels in order to differentiate between arteries and veins starting from the extrapulmonary arteries and veins.

### Statistical analysis

SPSS 26.0 software (SPSS) was performed for statistical analysis. Characteristics of the study population were compared by variance and Student *t* test for quantitative data or Mann-Whitney test and Fisher exact test for categorical variables. Correlations between mean log_jac values, pulmonary function indicators (VC%, FVC%, TLC%, and DLco%), and vascular-related parameters in IPF patients were analyzed by Spearman correlation coefficient, where absolute values of *r* < 0.2, 0.2–0.39, 0.40–0.59, 0.60–0.79, and > 0.8 were considered to have poor, weak, moderate, strong, and very strong correlation, respectively. A *p* value < 0.05 was considered statistically significant.

## Results

### Population characteristics

**Fig. 2:**
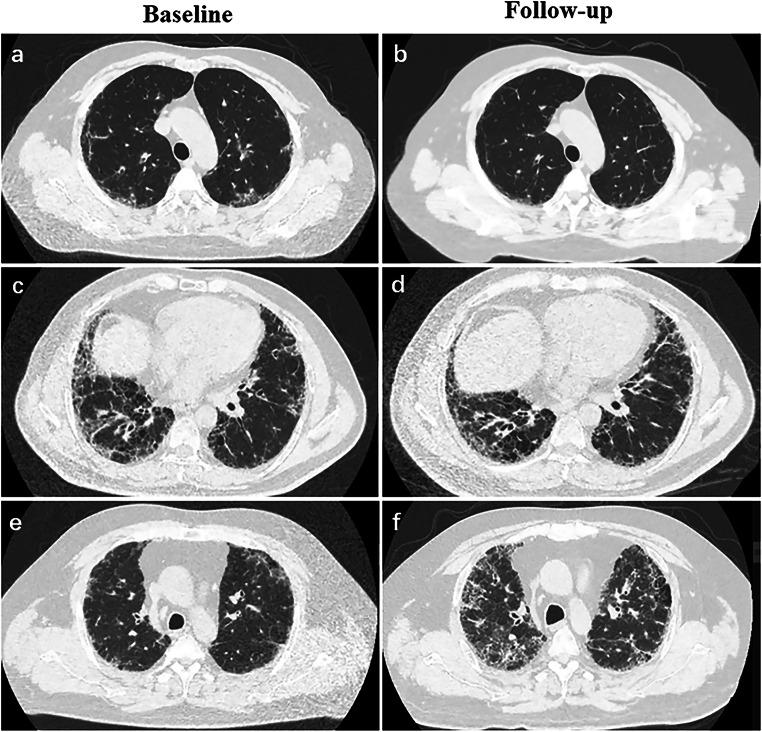
Morphological grouping based on visual assessment. **a**, **b** A 42-year-old patient with a significant reduction in the extent of the ground-glass opacity during 3 years of follow-up was classified in the group of visual improvement. **c**, **d** A 64-year-old patient with no significant change in the extent of the lung lesion during 6 months of follow-up was classified in the group of visual stability. **e**, **f** A 52-year-old patient with enlarged honeycombing, reticular pattern and ground-glass opacity during 18 months of follow-up was classified in the group of visual worsening

A total of 591 patients with IPF underwent HRCTs at China-Japan Friendship Hospital from January 2015 to May 2021. In total, 181 patients were excluded for the combination of other lung diseases. Patients who had motion artifacts on HRCTs (*n* = 21), patients who had no follow-up chest HRCT (*n* = 225), and patients who had no corresponding PFTs in the same day of HRCT (*n* = 95) failed to meet the inclusion criteria. Finally, 69 patients with IPF (male 66, median age 65 years, interquartile range (IQR), 60–70 years) were enrolled in this study (Fig. 1). Patients were divided into groups of functional stability (*n* = 44) and functional worsening (*n* = 25) according to PFTs. Moreover, patients were classified into groups of visual improvement (*n* = 4), visual stability (*n* = 21), and visual worsening (*n* = 44) based on HRCT (Examples of visual assessments are shown in Fig. 2).

At baseline, the median FVC% was 88.8% (IQR, 72.7–100.4%); 40.6% of patients (28 of 69) had a decreased FVC% (< 80% of the predicted value). There were no statistically significant differences in baseline PFT parameters (VC%, FVC%, TLC%, and DLco%) between the two groups of functional stability and functional worsening. The median DLco% was 51.4% (IQR, 36.3–66.0%) for the total population, and 77.0% of patients (53 of 69) had a decreased DLco% (< 70% of the predicted value). The median interval between baseline and follow-up HRCTs was 13.1months (IQR, 6.2–23.3 months). It was longer for patients with functional deterioration (median, 20.6 months [IQR, 11.7–24.8 months]) than for patients with functional stability (median, 9.0 months [IQR, 5.0–18.7 months]) (*p* = 0.012) (Table 1). There were also no statistically significant differences in baseline PFT parameters (VC%, FVC%, TLC%, and DLco%) between groups of visual stability and visual worsening (*p* > 0.05) (Table S1).

**Table 1:** Demographic and functional parameter characteristics of IPF patients based on pulmonary function tests

Characteristic	All patients (*n* = 69)	Patients with PFT stability (*n* = 44)	Patients with PFT worsening (*n* = 25)	*p* value
Median age (y)*	65 (60 to 70)	64 (60 to 68)	67 (60 to 72)	0.519
Male	66 (95.7)	42 (95.5)	24 (96.0)	0.915
Median baseline pulmonary function test values*				
VC%	85.6 (74.0 to 98.0)	88.0 (72.4 to 98.1)	78 (74.7 to 98.2)	0.819
TLC%	71.3 (61.6 to 78.5)	73.8 (61.2 to 78.6)	67.3 (62.1 to 80.5)	0.938
FVC%	88.8 (72.7 to 100.4)	89.8 (73.7 to 101.3)	78.3 (71.6 to 100.2)	0.704
DLco%	51.4 (36.3 to 66.0)	58.5 (48.1 to 69.5)	52.5 (44.0 to 67.3)	0.458
Median interval between baseline and follow-up chest CT (mo)*	13.1 (6.2 to 23.3)	9.0 (5.0 to 18.7)	20.6 (11.7 to 24.8)	0.012
Median changes in pulmonary function test results*				
VC%	−3.5 (−11.7 to 1.3)	−1.1 (−4.4 to 2.6)	−12.4 (−22.9 to −10.5)	< 0.001
TLC%	−3.3 (−7.7 to 2.0)	0.6 (−3.9 to 5.2)	−8.0 (−11.9 to −3.9)	< 0.001
FVC%	−4.6 (−12.0 to −1.7)	−0.7 (−5 to 3.1)	−14.0 (−21.7 to −11.0)	< 0.001
DLco%	−4.5 (−13.4 to 2.0)	−0.9 (−4.4 to 6.9)	−15.8 (−20.3 to −12.2)	< 0.001
Morphologic worsening at CT	44 (63.8)	21 (47.7)	23 (92.0)	0.001
Median mean log_jac*	0 (0 to 0)	0 (−0.02 to 0.02)	−0.10 (−0.61 to 0.02)	0.030

*IPF*, idiopathic pulmonary fibrosis; *PFT*, pulmonary function tests; *VC%*, percentage of predicted vital capacity; *FVC%*, percentage of predicted forced vital capacity; *TLC%*, percentage of predicted total lung capacity; *DLco%*, percentage of predicted diffusing capacity for carbon monoxide; *log_jac*, logarithm of the Jacobian determinant; *numbers in parentheses are the interquartile range. Figures in parentheses are percentages unless noted

**Fig. 3:**
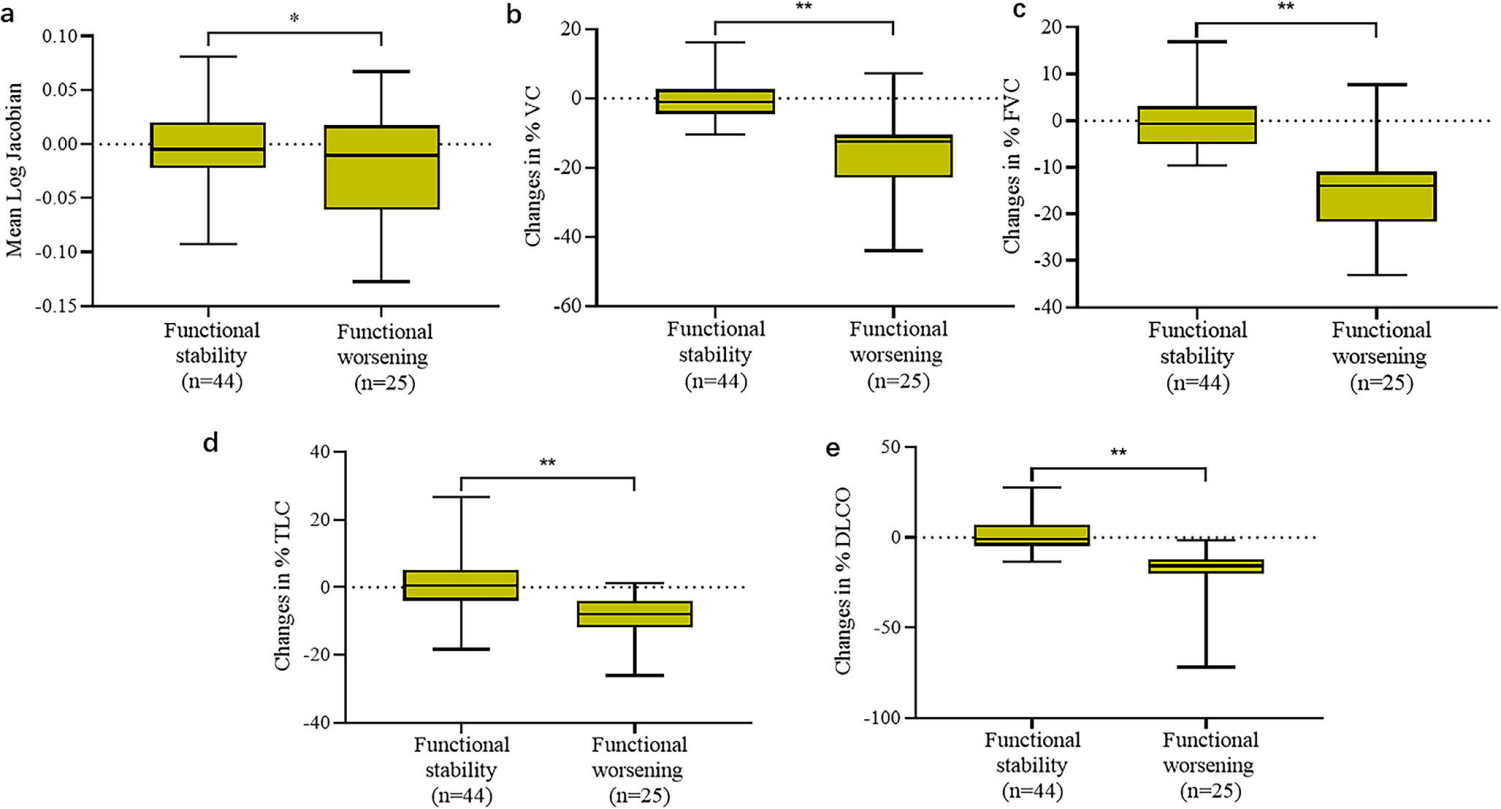
Box plots of the distribution of each parameter according to the functional assessment. The box plots show the characteristics of the data distribution of mean log_jac values, percentage of predicted vital capacity (VC%), forced vital capacity (FVC%), total lung capacity (TLC%), and predicted diffusing capacity for carbon monoxide (DLco%) between the two groups. *p < 0.05; **p < 0.001

In addition, there was a modest decline of lung function in the total population, with median changes of −4.6% (IQR, −12.0% to −1.7%) for FVC% and a median change of −4.5% (IQR, −13.4% to 2.0%) for DLco%. Nevertheless, there was a significant decrease in the population with functional deterioration compared to the population with functional stability with median changes of −14.0% (IQR, −21.7% to −11.0%) and −0.7% (IQR, −5.0% to 3.1%) for FVC% (*p* < 0.001), respectively, as well as −15.8% (IQR, −20.3% to −12.2%) and −0.9% (IQR, −4.4% to 6.9%) for DLco% (*p* < 0.001), respectively (Table 1 and Fig. 3). However, no statistically significant differences (*p* > 0.05) were observed in changes of PFT parameters (FVC%, TLC%, and DLco%) between groups of visual stability and visual worsening, except for VC% (*p* = 0.014) (Table S1). Visual deterioration occurred more often in patients with functional deterioration (23/25 [92.0%]) compared to patients with functional stability (21/44 [47.7%]) (*p* = 0.001) (Table 1).

**Fig. 4:**
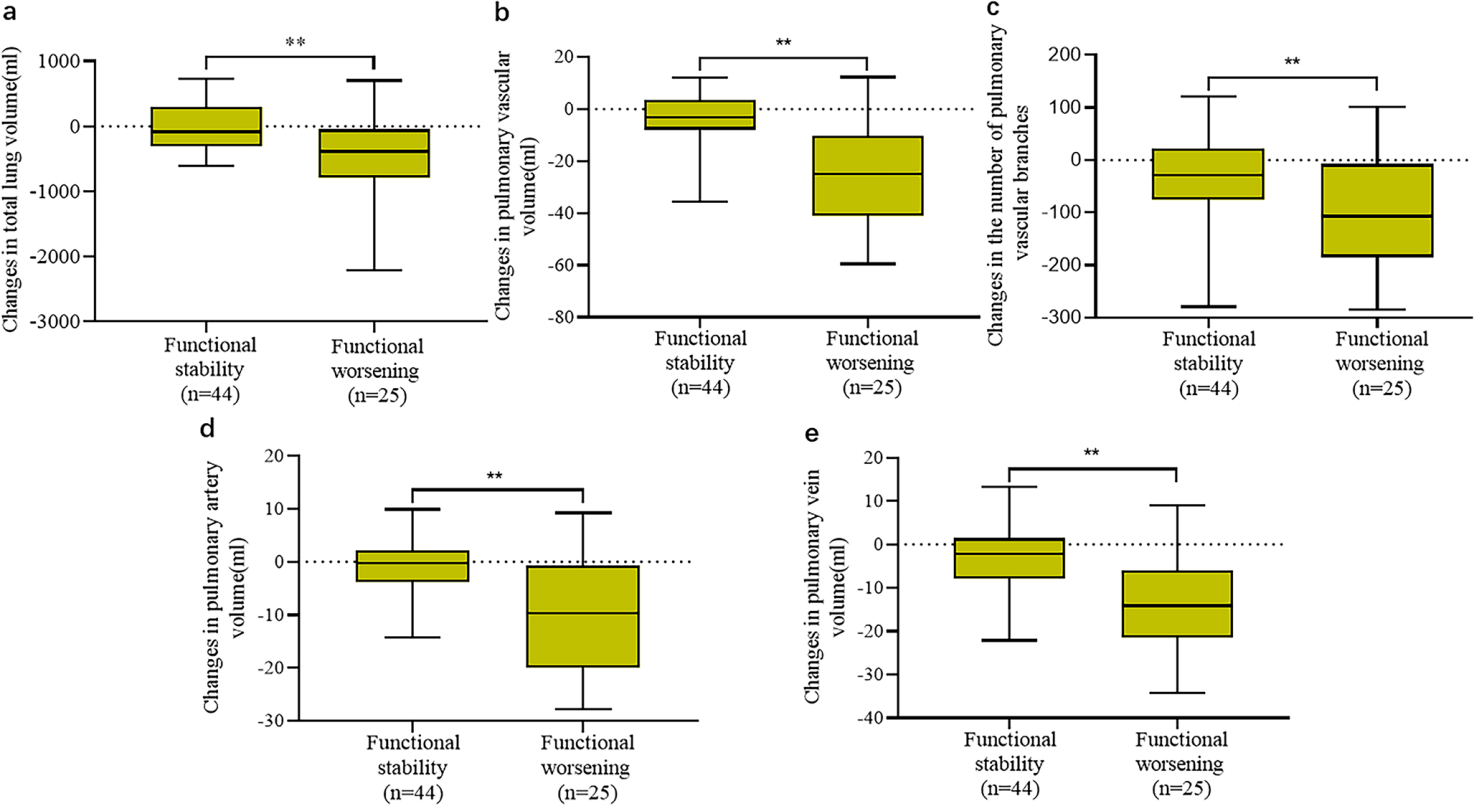
Box plots of the distribution of each parameter according to the functional assessment. The box plots show the characteristics of the data distribution of changes in total lung volume, pulmonary vascular volume, the number of pulmonary vascular branches, pulmonary artery volume, and pulmonary vein volume between the two groups

There were significant differences between patients with functional stability and those with functional deterioration in changes of median total lung volume: −85.9 mL (IQR, −302.1 mL to 302.8 mL) vs −385.2 mL (IQR, −796.5 mL to −45.2 mL), respectively (*p* = 0.01); changes in median pulmonary vascular volume −3.2 mL (IQR, −7.9 mL to 3.5 mL) vs −25.0 mL (IQR, −41.1 mL to −10.3 mL), respectively (*p* < 0.001); and changes in pulmonary artery volume −0.3 mL (IQR, −3.8 ml to 2.2 mL) vs −9.6 mL (IQR, −20.0 mL to −0.7 mL) , respectively (*p* < 0.001). Moreover, changes in the number of pulmonary vascular branches were significantly different between the two groups (*p* < 0.001) (Table 2 and Fig. 4). Nevertheless, there were no statistical differences in changes of total lung volume, pulmonary vascular volumes, and the number of pulmonary vascular branches between patients with functional stability and those with functional deterioration (*p* > 0.05) (Table S2). Additionally, baseline pulmonary function indicators including FVC%, VC%, TLC%, and DLco% were all correlated with pulmonary vascular volume (*p* < 0.01) and the number of vascular branches (*p* < 0.01).

**Table 2:** Lung volume and pulmonary vascular characteristics of IPF patients based on pulmonary function tests

Characteristic	All patients (*n* = 69)	Patients with PFT stability (*n* = 44)	Patients with PFT worsening (*n* = 25)	*p* value
Median pulmonary vascular–related indexes*				
Total lung volume (mL)	3671.1 (2979.1 to 4412.8)	3641.8 (3036.2 to 4540.2)	3700.3 (2838.8 to 4356.8)	0.942
Pulmonary vascular volume (mL)	86.1 (65.7 to 106.1)	91.4 (66.7 to 109.2)	80.7 (64.5 to 102.8)	0.413
The number of pulmonary vascular branches	422.0 (342.0 to 482.0)	423.5 (351.3 to 483.5)	419.0 (323.5 to 484.5)	0.394
Pulmonary artery volume (mL)	44.5 (34.4 to 53.9)	47.4 (33.5 to 60.4)	43.8 (34.4 to 49.9)	0.282
Pulmonary vein volume (mL)	40.2 (30.6 to 52.7)	36.3 (28.2 to 49.8)	36.6 (28.7 to 54.7)	0.598
Median changes in pulmonary vascular–related indexes*				
Total lung volume (mL)	−175.6 (−427.6 to 164.8)	−85.9 (−302.1 to 302.8)	−385.2 (−796.5 to −45.2)	0.010
Pulmonary vascular volume (mL)	−6.9 (−21.3 to 1.9)	−3.2 (−7.9 to 3.5)	−25.0 (−41.1 to −10.3)	< 0.001
The number of pulmonary vascular branches	−45.0 (−109.0 to 15.5)	−28.5 (−76.0 to 21.8)	−107.0 (−185.0 to −7.5)	0.005
Pulmonary artery volume (mL)	−2.3 (−8.9 to 2.0)	−0.3 (−3.8 to 2.2)	−9.6(−20.0 to −0.7)	< 0.001
Pulmonary vein volume (mL)	−5.8 (−14.7 to 0.4)	−2.3 (−7.8 to 1.4)	−14.1 (−21.5 to −6.0)	< 0.001

*IPF*, idiopathic pulmonary fibrosis; *PFT*, pulmonary function tests. *Numbers in parentheses are the interquartile range

### Elastic registration

The mean log_jac value was 0 (IQR, −0.02 to 0.02) in patients with functional stability, which was higher than those with functional deterioration of −0.10 (IQR, −0.61 to 0.02) (*p* = 0.03) (Table 1). Moreover, mean log_jac values were weakly positively correlated with VC% (*r* = 0.394, *p* < 0.05) and FVC% (*r* = 0.395, *p* < 0.05), and were moderately correlated with pulmonary vascular volume (*r* = 0.438, *p* < 0.01), especially pulmonary artery volume (*r* = 0.530, *p* < 0.01).

**Fig. 5:**
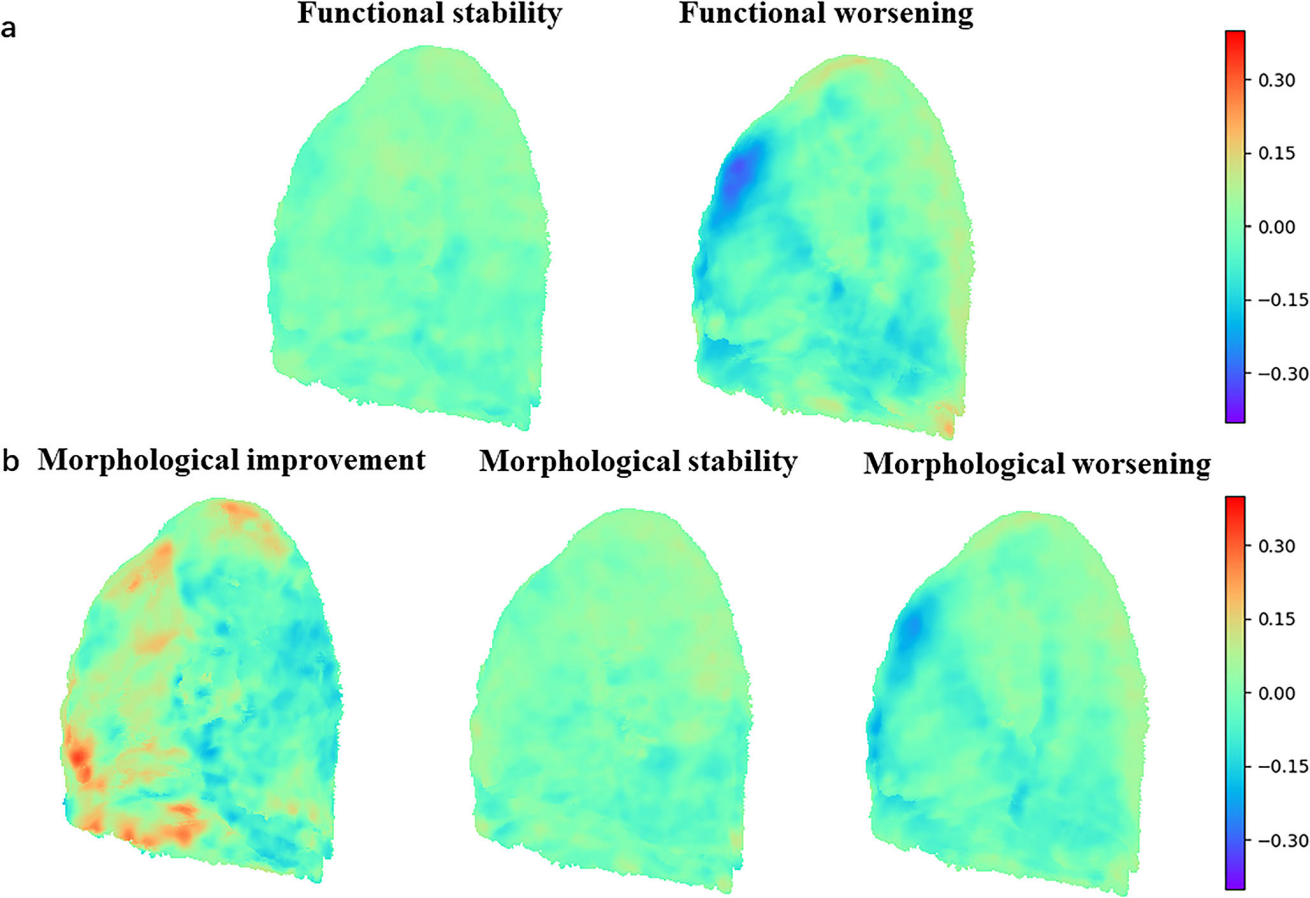
Jacobian map sagittal plane. Divided into two groups based on functional changes (**a**) and three groups based on morphological changes (**b**). **p < 0.001

**Fig. 6:**
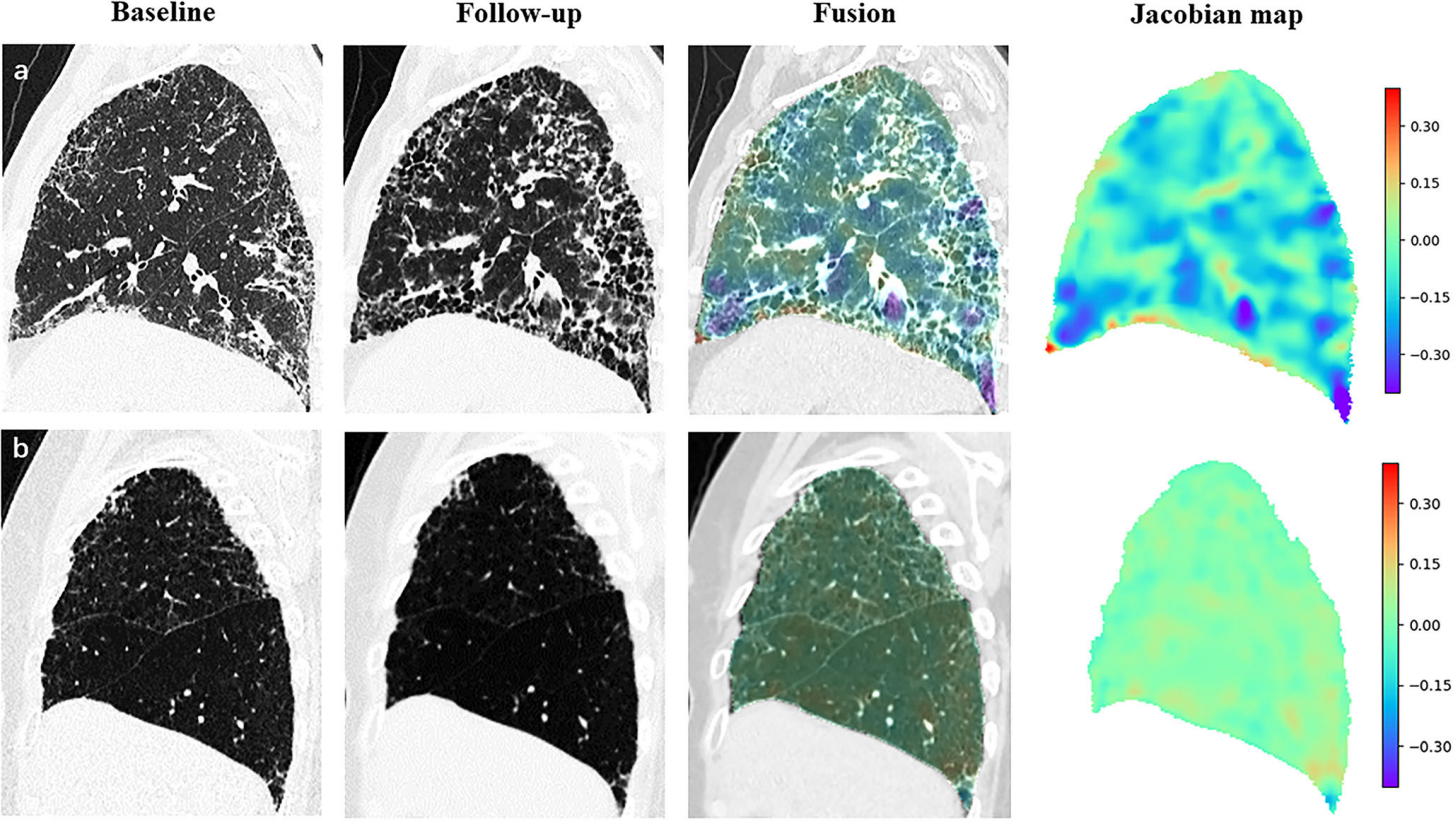
The representative Jacobian maps in two patients with IPF. **a** A 63-year-old patient with a follow-up interval of 30 months. Follow-up CT showed a significant increase in honeycombing and reticulations compared to baseline CT. Both visual and functional assessments supported disease progression. A Jacobian map showed diffuse lung shrinkage in correspondence with areas of increased fibrotic lesions and with a negative mean log_jac value. **b** A 77-year-old patient with 7-month follow-up interval. Follow-up CT showed no significant change in the lesions compared to baseline CT. Both visual and functional assessments indicated disease stability and the mean log_jac value was 0

Jacobian maps based on the distribution of log_jac values proved to be significantly different among various groups. Visualization of the sagittal plane showed that log_jac values were homogeneous in both groups of functional and morphologic stability (Fig. 5). In contrast, in patients with functional or morphologic deterioration, significant lung constriction was found in the lung base (negative log_jac values), while expansion was observed in the same part for patients with morphologic improvement (positive log_jac values). Baseline and follow-up HRCTs and Jacobian maps of patients with disease progression and disease stability are presented in Fig. 6.

## Discussion

We demonstrated, for the first time, the validity of a HRCT-based elastic registration technique to quantitatively assess the progression of IPF. Pulmonary fibrosis leads to pulmonary shrinkage, which is also an important manifestation of disease progression [18]. This shrinkage on HRCT is relatively difficult to assess visually. Elastic registration allows quantification of lung deformation and visualization in the form of color maps. In patients with functional and morphologic deterioration, a significant contraction of the base of the lung with negative mean log_jac values was observed. This is the preferred site of IPF lesions [19], but no such presentation was seen in patients with functional or morphologic stability. Also, a significant difference in the mean log_jac values can be seen between patients with functional stability and deterioration (*p* < 0.05).

PFTs have been most frequently used to assess disease progression in patients with IPF [17, 20]. In our study, PFTs including VC%, FVC%, TLC%, and DLco% were all statistically different in both groups of patients (*p* < 0.01). However, PFTs describe the overall lung status, whereas elastic registration can visualize the changes in local lesions. Chassagnon et al [12] have described the value of elastic registration in assessing disease progression during the follow-up of SSc-ILD, but no relevant studies have explored the role of elastic registration in the progression of IPF. Several researches have demonstrated that vessel-related structures are superior to visual scores and functional parameters in predicting prognosis in multiple diseases. However, the exact mechanism has not been elucidated. The strong correlation reported between pulmonary vascular volumes and PFTs suggested the potential of vessel-related structures to be an important new index for assessing disease severity in patients with IPF [5, 6, 21–24]. Similar results are also observed in our research. Additionally, it is interesting to note that our study found that mean log_jac values were also correlated with the changes of pulmonary vascular volume (*r* = 0.438, *p* < 0.01), especially pulmonary artery volume (*r* = 0.530, *p* < 0.01), and were positively correlated with the changes in the number of pulmonary vascular branches (*r* = 0.326, *p* < 0.01), suggesting that lung fibrosis, which causes lung shrinkage, also disrupts the pulmonary vasculature accompanied by a decrease in vascular volume and number, and is also associated with disease progression. In summary, the results indicated that mean log_jac values based on elastic registration may be used to assess the progression of IPF during follow-up.

There are still several limitations. First, a number of patients who died or lost to follow-up at our institution or who were unable to complete PFTs due to exacerbations, and cases who were unable to achieve flexible alignment due to poor CT quality or co-infection were excluded. The fewer cases included were the main reason leading to the weak correlation. Second, this was a retrospective study with an uneven range of time intervals between baseline and follow-up HRCTs. The interval was longer for patients with disease deterioration than for those with disease stability. Third, the degree of inspiration of patients cannot be well controlled due to the nature of the retrospective study, which may lead to uneven image quality and even have an impact on our results. Because of our very strict design of included patients, many patients who lacked high-quality and longitudinal HRCT data or did not have HRCT and PFTs on the same day were excluded. Although the number of patients included was not large enough to draw reliable conclusions, the results suggest that this approach is feasible. In further research, we will conduct a multicenter study to validate the value of the elastic registration technique in the assessment of IPF.

## Conclusions

Lung elastic shrinking evaluated with an elastic registration technique on HRCT is a novel imaging biomarker in the quantitative analysis of IPF.

## Supplementary Information

ESM 1 (DOCX 50 kb)

## Abbreviations

DLco%: Percentage of predicted diffusing capacity for carbon monoxide
FVC%: Percentage of forced vital capacity
HRCT: High-resolution computed tomography
ILD: Interstitial lung disease
IPF: Idiopathic pulmonary fibrosis
PFTs: Pulmonary function tests
SSc-ILD: Systemic sclerosis–associated interstitial lung disease
TLC%: Percentage of total lung capacity
VC%: Percentage of predicted vital capacity

## Declarations

### Guarantor

The scientific guarantor of this publication is Prof. Huaping Dai, MD.

### Statistics and biometry

No complex statistical methods were necessary for this paper.

### Methodology

• retrospective

• diagnostic study

• performed at one institution
