# Supplementary material for: Lung shrinking assessment on HRCT with elastic registration technique for monitoring idiopathic pulmonary fibrosis
Source: Eur Radiol. 2022 Nov 23;33(4):2279–88. doi: 10.1007/s00330-022-09248-7 (PMC10017651; doi:10.1007/s00330-022-09248-7)
Supplement: Supplementary file 1 — (DOCX 50 kb) [file 330_2022_9248_MOESM1_ESM.docx]

**Table S1** Demographic and functional parameter characteristics of IPF patients based on visual assessment on HRCT

| Characteristic | All Patients  (*n* = 69) | Patients with Visual  Improvement on HRCT (*n* = 4) | Patients with Visual  Stability on HRCT  (*n* = 21) | Patients with Visual  Worsening on HRCT  (*n* = 44) | *p* Value |
| --- | --- | --- | --- | --- | --- |
| Median age (y)* | 65(60 to 70) | 59(46 to 66) | 62(57 to 68) | 66(63 to 70) | 0.155 |
| Male | 66(95.7) | 4(100.0) | 19(90.5) | 43(97.7) | 0.193 |
| **Median baseline PFT values*** |  |  |  |  |  |
| VC% | 85.6(74.0 to 98.0) | 89.7(78.5 to 100.7) | 74.6(65.9 to 90.7) | 88.1(75.5 to 98.8) | 0.082 |
| TLC% | 71.3(61.6 to 78.5) | 73.7(71.6to 80.0) | 63.2(60.2 to 75.1) | 74.5(63.7 to 81.1) | 0.050 |
| FVC% | 88.8(72.7 to 100.4) | 93.5(79.7 to 102.9) | 76.7(67.5 to 90.7) | 91.1(76.2 to 101.5) | 0.055 |
| DLco% | 51.4(36.3 to 66.0) | 46.8(38.8 to 61.1) | 56.9(49.8 to 66.8) | 57.1(44.0 to 73.1) | 0.931 |
| Median interval between baseline and follow-up HRCT (mo)* | 13.1(6.2 to 23.3) | 18.2(7.4 to 32.8) | 6.2(3.1 to 10.7) | 19.6(9.0 to 24.4) | <0.001 |
| **Median changes in PFT*** |  |  |  |  |  |
| VC% | -3.5(-11.7 to 1.3) | -3.2(-10.2 to 6.3) | -1.4(-5.1 to 1.2) | -4.8(-13.6 to -1.1) | 0.014 |
| TLC% | -3.3(-7.7 to 2.0) | 1.9(-2.6 to 6.5) | 0.3(-5.2 to 2.4) | -4.2(-10.1 to -1.0) | 0.185 |
| FVC% | -4.6(-12 to -1.7) | -2.8(-9.4 to 5.7) | -1.3(-6.5 to 2.4) | -6.1(-14.8 to 0.8) | 0.019 |
| DLco% | -4.5(-13.4 to 2.0) | 6.1(-6.8 to 19.4) | -2.1(-4.2 to 4.0) | -8.8(-15.7 to -0.5) | 0.185 |
| Morphologic worsening at HRCT | 44(63.8) | 0(0) | 2(9.5) | 23(52.3) | 0.001 |
| Median mean log_jac* | 0(0 to 0) | 0(-0.02 to 0.04) | -0.01(-0.02 to 0.01) | 0(-0.04 to 0.02) | 0.346 |

IPF, idiopathic pulmonary fibrosis, PFT: pulmonary function tests; HRCT, high-resolution computed tomography; VC%, percentage of predicted vital capacity; FVC%, percentage of predicted forced vital capacity; TLC%, percentage of predicted total lung capacity; DLco%, percentage of predicted diffusing capacity for carbon monoxide; log_jac, logarithm of the Jacobian determinant; * Numbers in parentheses are the interquartile range. Figures in parentheses are percentages unless noted. P values were used to compare the differences between groups for visual stability and visual worsening.

**Table S2** Lung volume and pulmonary vascular characteristics based on visual assessment on HRCT

| Characteristic | All Patients (*n* = 69) | Patients with Visual  Improvement on HRCT  (*n* = 4) | Patients with Visual  Stability on HRCT  (*n* = 21) | Patients with Visual  Worsening on HRCT  (*n* = 44) | *p* Value |
| --- | --- | --- | --- | --- | --- |
| **Median pulmonary vascular-related indexes*** |  |  |  |  |  |
| Total lung volume (ml) | 3671.1(2979.1 to 4412.8) | 3887.7(2817.4 to 49338.9) | 3408.2(2906.5 to 4050.8) | 3919.4(3321.9 to 4626.0) | 0.090 |
| Pulmonary vascular volume (ml) | 86.1(65.7 to 106.1) | 80.0(55.1 to 114.5) | 68.9(52.5 to 95.9) | 89.4(73.2 to 108.4) | 0.025 |
| The number of pulmonary vascular branches | 422.0(342.0 to 482.0) | 374.0(278.8 to 451.3) | 371.0(302.5 to 456.5) | 429.0(363.0 to 500.0) | 0.061 |
| Pulmonary artery volume (ml) | 44.5(34.4 to 53.9) | 40.3(30.8 to 58.6) | 39.1(30.1 to 52.9) | 46.8(39.5 to 54.0) | 0.070 |
| Pulmonary vein volume (ml) | 40.2(30.6 to 52.7) | 39.7(24.4 to 55.9) | 34.9(25.7 to 44.2) | 41.7(32.3 to 55.5) | 0.015 |
| **Median changes in pulmonary vascular-related indexes *** |  |  |  |  |  |
| Total lung volume (ml) | -175.6(-427.6 to 164.8) | -106.8(-366.6 to 351.0) | -37.4(-213.3 to 180.3) | -284.1(-584.7 to 180.3) | 0.518 |
| Pulmonary vascular volume (ml) | -6.9(-21.3 to 1.9) | -3.6(-15.2 to 6.0) | -2.5(-5.3 to 3.4) | -16.4(-30.4 to -2.4) | 0.944 |
| The number of pulmonary vascular branches | -45.0(-109.0 to 15.5) | -29.0(-44.5 to 13.5) | 9.0(-48.5 to 21.0) | -92.5(-141.0 to -5.0) | 0.785 |
| Pulmonary artery volume (ml) | -2.3(-8.9 to 2.0) | 1.3(-5.5 to 7.9) | -0.3(-2.5 to 2.7) | -5.4(-12.4 to 1.7) | 0.789 |
| Pulmonary vein volume (ml) | -5.8(-14.7 to 0.4) | -6.3(-15.2 to 5.0) | -1.7(-4.6 to 1.5) | -10.9(-17.8 to -2.3) | 0.888 |

HRCT, high-resolution computed tomography. * Numbers in parentheses are the interquartile range. P values were used to compare the differences between groups for visual stability and visual worsening.
